# Diagnosis and treatment of isolated autosomal recessive woolly hair/hypotrichosis

**DOI:** 10.3389/fmed.2025.1605851

**Published:** 2025-12-05

**Authors:** Ying Xie, Sha Luo, Yumei Yang, Xin Zou, Shuying Lv, Meijiao Du, Yonglong Xu, Xiaojuan Song, Changjie Qi, Nuo Li, Dingquan Yang

**Affiliations:** 1 Graduate School, Beijing University of Chinese Medicine, Beijing, China; 2 Department of Dermatology, The National Center for the Integration of Traditional Chinese and Western Medicine, China-Japan Friendship Hospital, Beijing, China; 3 Department of Dermatology, The First Affiliated Hospital of Hunan University of Chinese Medicine, Changsha, Hunan, China; 4 School of Senior Translation College, Dalian University of Foreign Languages, Dalian, Liaoning, China; 5 Department of Rehabilitation Medicine, The 8th Medical Center, PLA General Hospital, Beijing, China

**Keywords:** autosomal recessive woolly hair, diagnostic, genetic, treatment, ARWH

## Abstract

Isolated autosomal recessive woolly hair/hypotrichosis (ARWH, OMIM:278150) is a rare congenital disorder marked by sparse, tightly curled “woolly” hair. ARWH is associated with mutations in *LIPH*, *LPAR6*/P2RY5, *KRT25*, and *C3ORF52*, with *LIPH* and *LPAR6* as the primary causative genes. Mutation prevalence varies globally: in Japan, founder mutations c.736 T > A (p.Cys246Ser) and c.742C > A (p.His248Asn) in *LIPH* are predominant; Pakistan reports a recurrent *LIPH* exon 5 deletion (c.659_660del); Russia’s Volga-Ural region has an exon 4 deletion (c.527_628del); and 12 out of 19 Chinese ARWH cases are linked to *LIPH* c.742C > A. *LPAR6* mutations are sporadic, with rare occurrences in Pakistani families and two Chinese cases. *KRT25* mutations include the Russian founder variant c.712G > T (p.Val238Leu) and the Pakistani c.950 T > C (p.Leu317Pro). *C3ORF52* mutations are newly identified and reported only in two U. S. cases. No definitive treatment exists, but minoxidil, gentamicin, regenerative therapies, and plant-derived compounds show potential. Regional mutation patterns highlight genetic founder effects and population-specific variations in ARWH pathogenesis.

## 1 Introduction

Isolated autosomal recessive woolly hair/ hypotrichosis (ARWH, OMIM:278150) is a rare congenital hair abnormality that typically manifests at birth or within the first 2 years of life (1). Clinically, it presents as sparse, thin, and tightly curled hair, resembling sheep’s wool, which may be accompanied by reduced hair pigmentation and increased fragility. Based on presentation and distribution, it can be classified as generalized (affecting the entire scalp and body), localized (confined to a scalp area as a woolly hair nevus), or diffuse partial (primarily occurring in adolescence and adulthood). This condition can occur in isolation or as part of a genetic syndrome and is thus divided into syndrome and non-syndrome forms (2).

In 1907, Gossage first documented this phenomenon in a European family (3). In 1974, Hutchinson et al. classified non-syndromic woolly hair into three types based on genetic characteristics: (1) woolly hair nevus (non-hereditary, OMIM:194050), (2) autosomal dominant woolly hair (ADWH, hereditary, OMIM:194040), and (3) autosomal recessive woolly hair (ARWH, familial, OMIM:278150). Woolly hair nevus presents as well-circumscribed scalp woolly patches (rarely elsewhere) without systemic abnormalities or gene mutations, stabilizing post-childhood without extensive alopecia. Autosomal dominant (ADWH) involves generalized woolly hair (entire scalp, often eyebrows) persisting into adulthood, linked to *KRT74* mutations but rarely causing severe hypotrichosis (normal hair density with only texture issues). Autosomal recessive (ARWH) onsets at birth or early infancy, featuring generalized sparse, tightly curled woolly hair and progressive hypotrichosis (age-related thinning), mainly caused by mutations in *LIPH*, *LPAR6* (*P2RY5*), *KRT25*, or *C3ORF52* (4). Localized autosomal recessive hypotrichosis (LAH) differs from ARWH by focal alopecia (mostly in the scalp parietal region or eyebrows, no generalized woolly hair) and is specifically associated with *C3ORF52* mutations. This article reviews and summarizes the literature on the clinical manifestations, diagnosis, genetics, and treatments of isolated ARWH, providing insights for clinical practice.

## 2 Materials and methods

This study explored the clinical manifestations, diagnosis, genetic pathogenesis, and treatment of ARWH through a comprehensive literature search. Relevant literature was retrieved from databases including CNKI, Wanfang Data, VIP Chinese Journal Database, and PubMed. The search terms used were (“Autosomal Recessive Woolly Hair” OR “ARWH” OR “Woolly Hair Hypotrichosis”), with a publication cutoff of December 2024.

Literature was included based on the following criteria:

1. Publicly published literature with accessible and readable full texts;
2. Original studies, case reports, case series, or reviews focusing on the clinical manifestations, genetic characteristics, diagnosis, or treatment of ARWH;
3. For case reports, clear genetic mutation information must be provided.

To ensure the reliability and validity of the included evidence, the following types of literature were excluded during the screening process:

1. Duplicate reports.
2. Literature on diseases presenting with woolly hair but not ARWH.
3. Undiagnosed case reports.

Finally, 63 English articles and 22 Chinese articles were included, consisting of 63 case reports and 7 reviews.

## 3 Discussion

### 3.1 Clinical manifestations of isolated ARWH

**Figure 1:**
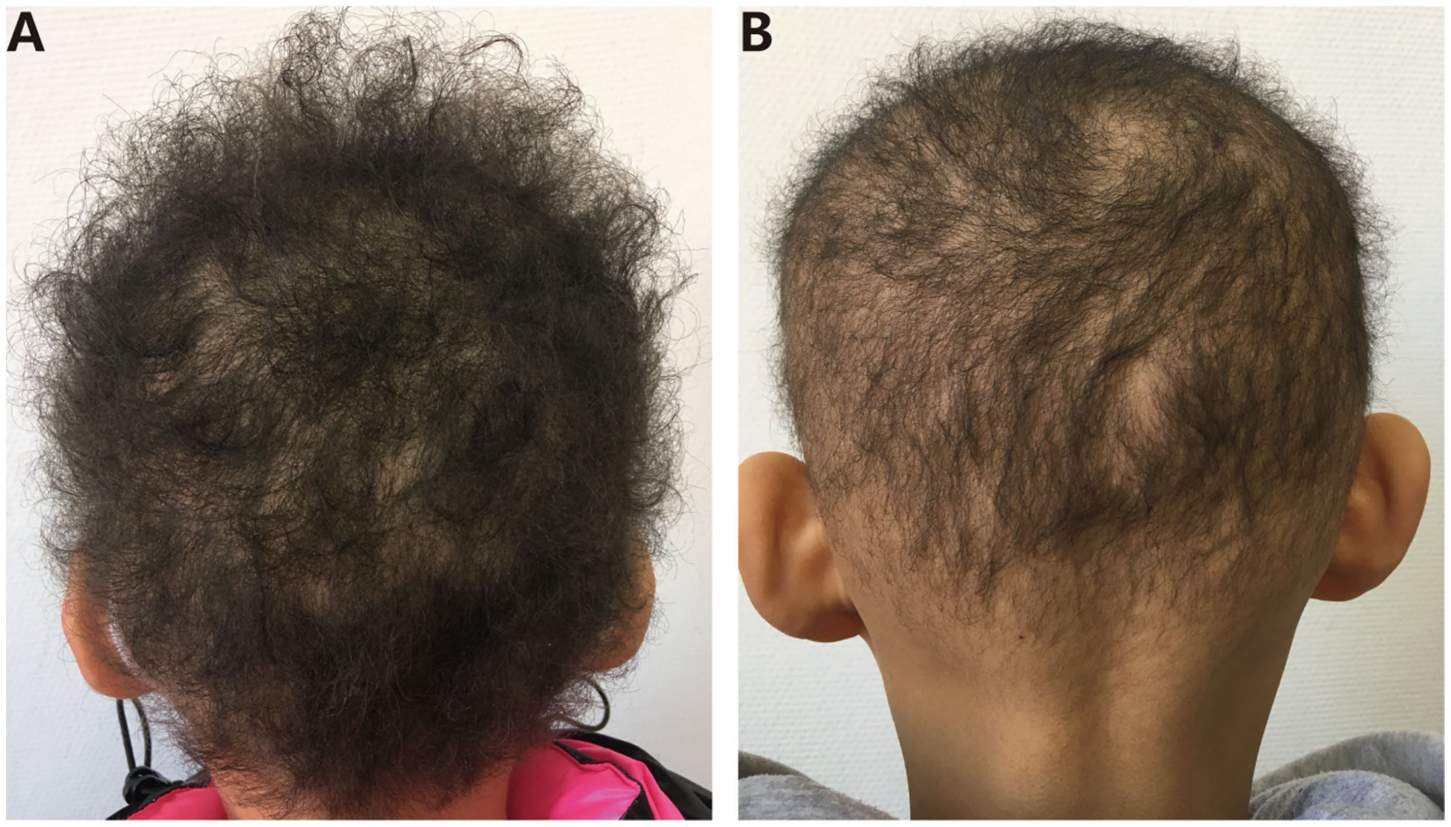
**(A,B)** ARWH patient’s clinical manifestations; the patient’s hair is curly or wavy, with a “sheep’s wool appearance,” and there are varying degrees of hair thinning.

ARWH is a rare structural abnormality of the hair shaft with limited growth potential. The main clinical features include abnormal hair characteristics at birth, slow hair growth, and cessation of growth after reaching a certain length (rarely exceeding 12 cm) (4). Hair across the entire scalp is coarse, dry, and lusterless, with spiral or wavy curls resembling scattered sheep’s wool, accompanied by varying degrees of sparseness, increased fragility, and reduced pigmentation. The majority of patients have normal eyebrows, eyelashes, beards, armpit hair, pubic hair, nails, teeth, and sweating function, with rare cases of palmoplantar keratoderma and perifollicular keratosis. Histopathological examination of scalp tissue reveals abnormalities in hair follicles and hair shafts. Most patients have varying degrees of hypotrichosis, with alopecia totalis as the most severe form. Hair condition may improve or worsen with age (5) (Figure 1).

### 3.2 Microscopic, scanning electron microscopy (SEM), and trichoscopy characteristics of isolated ARWH

#### 3.2.1 Microscopy

Plucked hairs from affected individuals exhibit a wavy appearance, with most showing signs of nutritional deficiency and absence of root sheath components at the hair bulb.

#### 3.2.2 SEM

Under SEM, ARWH presents the following characteristics (4): (1) flat shape, with oval or irregular cross-section; (2) longitudinal and transverse grooves on the proximal hair shaft, with normal cortical cell striations but worn free edges; (3) uneven and irregular twisting on both proximal and distal hair shafts (distinct from true twisted hair); and (4) possible absence or damage of distal cortical cells leading to hair breakage, presenting as nodular fragile hair, trichorrhexis nodosa, or trichoschisis.

#### 3.2.3 Trichoscopy

**Figure 2:**
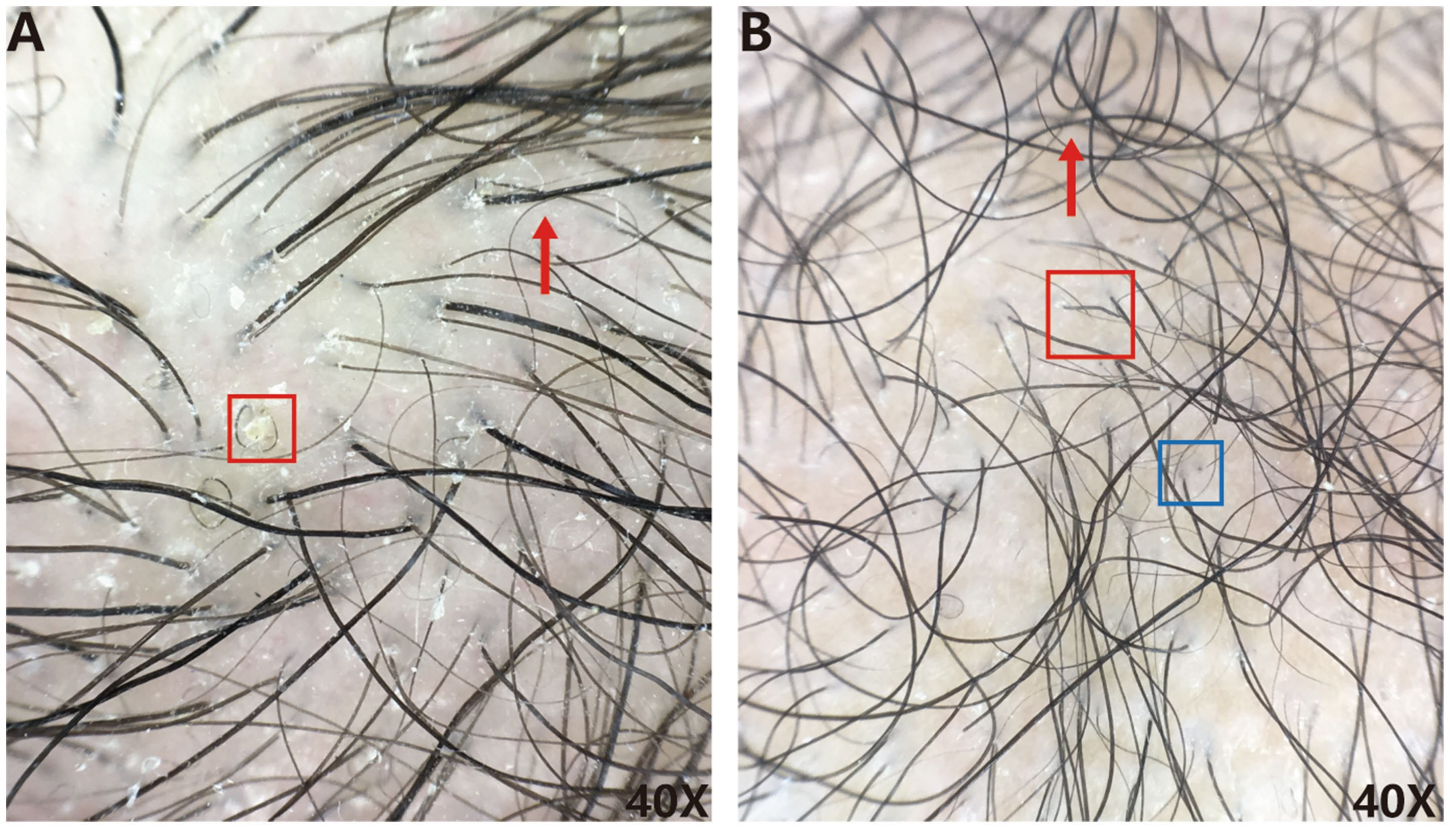
**(A,B)** Corresponding hair microscopy findings of two ARWH patients **(A)** curly hair (red box), wavy hair (red arrow), **(B)** black spots sign (blue box), broken hair (red box), and wavy hair (red arrow).

Trichoscopic examination reveals intensely wavy, serpentine hairs accompanied by hair shaft breakage. While not essential for diagnosis, the typical wavy hair appearance is suggestive of ARWH and warrants further clinical evaluation (Figure 2).

### 3.3 The diagnosis and differential diagnosis of isolated ARWH

The diagnosis of ARWH requires a combination of clinical manifestations and genetic testing, with differentiation from other congenital hair shaft abnormalities. Although ARWH often occurs in isolation, it is crucial to rule out syndrome forms, including Carvajal syndrome (OMIM:605676), Naxos disease (OMIM:601214), ectodermal dysplasia-skin fragility syndrome (ED-SF syndrome, OMIM:604536), tricho-hepato-enteric syndrome (TDOS, OMIM:222470), and Menkes disease (MD, OMIM:309400). Syndromic woolly hair is often characterized by multisystem involvement, frequently accompanied by epidermal and dermal symptoms. For newborns with woolly hair, differentiation can be achieved by assessing for additional systemic manifestations (e.g., hair keratotic disorders, palmoplantar keratoderma, and cardiac or sensory abnormalities) combined with family history and genetic testing.

Carvajal syndrome is mainly characterized by woolly hair, striate palmoplantar keratoderma, and left-sided dilated cardiomyopathy (6). Naxos disease is a right ventricular arrhythmogenic cardiomyopathy associated with diffuse palmoplantar keratoderma and woolly hair. Both conditions are inherited in an autosomal recessive manner, with Naxos disease caused by *DSG2* mutations and Carvajal syndrome caused by *DSC2* mutations (7). ED-SF syndrome, an autosomal recessive disorder caused by *PKP1* mutations, presents with fragile skin, chronic lip inflammation, palmoplantar keratoderma, abnormal hair growth, and nail dystrophy (8). TDOS, caused by dominant *DLX3* mutations, is an ectodermal development disorder characterized by dental abnormalities (e.g., enamel hypoplasia and malocclusion), cranial sclerosis, and hair/nail anomalies (2). MD (also known as curly hair syndrome) is a rare X-linked recessive disorder caused by *ATP7A* mutations, featuring markedly wavy hair, progressive neurodegeneration, and connective tissue abnormalities (9). Additionally, woolly hair can also be seen in rarer syndromes such as Noonan syndrome, cardiofaciocutaneous syndrome, and Costello syndrome, all of which share features of growth retardation, cardiac defects, intellectual disability, and embryonic development abnormalities (Table 1).

**Table 1:** Key syndromic woolly hair disorders.

Syndrome	Clinical “Red Flags”	Inheritance pattern	Causal gene(s)
Carvajal syndrome	Woolly hair, striate palmoplantar keratoderma, left-sided dilated cardiomyopathy	Autosomal Recessive	DSC2
Naxos disease	Woolly hair, diffuse palmoplantar keratoderma, right ventricular arrhythmogenic cardiomyopathy	Autosomal Recessive	DSG2
ED-SF syndrome	Woolly/abnormal hair, fragile skin, chronic lip inflammation, palmoplantar keratoderma, nail dystrophy	Autosomal Recessive	PKP1
TDOS	Dental abnormalities (enamel hypoplasia, malocclusion), woolly/abnormal hair, cranial sclerosis, nail anomalies	Autosomal Dominant	DLX3
MD	Curly (woolly appearing) hair, progressive neurodegeneration, connective tissue defects	X-linked Recessive	ATP7A

Non-syndromic hair shaft abnormalities requiring differentiation include monilethrix, pili torti, trichorrhexis nodosa, pseudopili annulati, and trichothiodystrophy. Monilethrix is identified by periodically narrowed hair shafts, prone to breakage at these narrow sections (10), often accompanied by perifollicular papules and erythema (absent in ARWH). Pili torti involves flattened hair shafts with 90°–360° curls along the axis, which are milder and irregularly spaced compared to ARWH (10). Trichorrhexis nodosa is characterized by longitudinal hair shaft breakage into multiple fibers, producing a “brush-like appearance” under dermatoscopy (11). Pseudopili annulati (bamboo hair) results from hair shaft invagination (stacking) at specific points.

### 3.4 Genetics of isolated ARWH

Advancements in molecular biology and statistical methods have enhanced the understanding of ARWH pathogenesis through two-point linkage analysis for pathogenic gene localization and cloning. Type 1 autosomal recessive woolly hair (ARWH1, OMIM:278150) is caused by *LPAR6 (P2RY5)* mutations, type 2 (ARWH2, OMIM:604379) by *LIPH* mutations, and type 3 (ARWH3, OMIM:616760) by *KRT25* mutations. In recent years, missense mutations in *C3ORF52* have been identified in ARWH patients from two independent families (4).

The *LIPH* gene, located on human chromosome 3q27.2, contains 10 exons and encodes a 451-amino acid membrane-bound triacylglycerol lipase. Its primary function is to catalyze phosphatidic acid (PA) hydrolysis to produce 2-acyl lysophosphatidic acid (LPA), an extracellular mediator that promotes hair growth (12). LPA is the ligand for the G protein-coupled receptor P2Y5, encoded by the *LPAR6* gene, which is expressed in the Henle, Huxley, and basement membrane layers of the inner hair root sheath. LIPH is also highly expressed in the Huxley layer of the inner hair sheath and outer hair root sheath, suggesting overlapping roles in hair follicle differentiation and maturation via the LIPH/LPA/P2Y5 pathway.

The *KRT25* gene encodes keratin 25, a member of the keratin family critical for cytoskeletal formation and maintenance in hair, skin, and nails. Mutations disrupt keratin 25 function, impairing hair cell stability and morphology, causing ARWH (13).

Chromosome 3 Open Reading Frame 52 (*C3ORF52*) is thought to be necessary for *LIPH*-mediated LPA synthesis, but its exact function remains unclear. Mutations may affect cell signaling or cytoskeletal structure during hair development, impacting hair morphology (14). Further research is needed to elucidate its specific mechanisms.

Since Kazantseva et al. identified the *LIPH* gene as a contributor to human hair development defects in 50 families from the Volga-Ural region of Russia in 2006, researchers have continuously explored the pathogenic genes and pathogenesis of ARWH. To date, over 30 *LIPH* mutations (Table 2), more than 20 *LPAR6* gene mutations (Table 3), and 2 mutations each in the *KRT25* and *C3ORF52* genes (Table 4) have been detected across different regions and ethnic groups. These mutations encompass frameshift, missense, and splice site variants, with homozygous mutations being the most prevalent, followed by compound heterozygous forms. In China, ARWH was first reported by Shen Dawei in 1985; in recent years, with the deepening understanding of woolly hair, 19 ARWH family pedigrees have been documented domestically.

**Table 2:** Reported *LIPH* gene mutations in international patients with ARWH.

Exon	Mutation	Variation ID/Accession (ClinVar)	Amino acid changes	Variant type	Ethnicity	Founder/Recurrent	Region	Reference
2	c.322 T > C	3,305/VCV000003305.1	p.Trp108Arg	Missense	Pakistani	Yes	South Asia	(22)
2	c.328C > T	280,138/VCV000280138.4	p.Arg110*	Nonsense	Pakistani	No	South Asia	(23)
2	c.346–350delATATA	/	FS/PTC120	Deletion	Pakistani	No	South Asia	(24)
2	c.280_369dup	3,306/VCV000003306.3	p.Gly94_Lys123dup	In-frame duplication	Pakistani, Israeli, Turkish, Arab, Jewish	Yes	South Asia, Middle East, Europe	(22, 25–28)
2	c.179C > G	/	p.Ser60*	Nonsense	Lebanese	No	South Asia	(29)
2	c.403_409dup	/	p.Gln137HisfsX1	Frame-shift	Austrian	No	Europe	(28)
3	c.460_461AG > GA	/	p.Ser154Asp	Missense	Japanese	No	East Asia	(30)
3	c.454G > A	/	p.Gly152Arg	Missense	Chinese	No	East Asia	(31)
4	c.624delT	/	FS/PTC217	Deletion	Pakistani	No	South Asia	(5)
4	c.558_559insT	/	p.Lys187Ter	Missense	Japanese	No	East Asia	(32)
4	c.527_628del	/	176G-209D(34aa)deletion	Deletion	Russian	Yes	Europe	(33)
4	c.614A > G	/	p.His205Arg	Missense	Japanese, Chinese	No	East Asia	(34, 35)
4	c.619G > C	/	p.Asp207His	Missense	Japanese	No	East Asia	(36)
4	c.530 T > G	/	p.Leu177Arg	Missense	Chinese	No	East Asia	(37)
5	c.659_660delTA	/	p.Ile220Argfs*29	Deletion	Pakistani, Guyanese	Yes	South Asia, South America	(22, 38)
5	c.682delT	/	FS/PTC259	Deletion	Pakistani	No	South Asia	(5)
5	c.688C > T	/	p.Gln230*	Nonsense	Pakistani	No	South Asia	(39)
5	c.686delAins18	/	p.Asp229Glyfs*22	Frame-shift	Japanese	No	East Asia	(40)
5	c.699C > G	/	p.Cys233Trp	Missense	Japanese	No	East Asia	(41)
5	c.671C > G	/	p.Pro224Arg	Missense	Japanese	No	East Asia	(42)
5	c.686delinsGTAGAACCCAACCTGGCT	3,775,180/VCV003775180.1	p.Asp229fs37X	Frame-shift	Chinese	No	East Asia	(43)
5	c.629-1_629delinsTT	/	Not reported	Splice	Chinese	No	East Asia	(44)
6	c.778A > T	/	p.Arg260X	Nonsense	Pakistani	No	South Asia	(22)
6	c.736 T > A	225,403/VCV000225403.9	p.Cys246Ser	Missense	Japanese, Chinese	Yes	East Asia	(32, 45)
6	c.742C > A	30,669/VCV000030669.6	p.His248Asn	Missense	Japanese, Chinese	Yes	East Asia	(30, 46)
7	c.932delC	/	p.Pro311Leufs*3	Deletion	Pakistani	No	South Asia	(47)
7	c.973C > T	/	p.Pro325Ser	Missense	Chinese	No	East Asia	(48)
7	c.982 + 12A > G	/	Not reported	Splice	Chinese	No	East Asia	(44)
7, 8	Ex7_8del	/	FS/PTC297	Deletion	Pakistani, Guyanese	Yes	South Asia, South America	(5, 49)
10	c.1303_1309dupGAAAACG	/	Val437GlyfsX4	Insertion	Guyanese	No	South America	(38)
Intron 2	c.417 + 1G > C	/	Not reported	Splice	Japanese	No	East Asia	(50)
Intron 4	c.620_627delACACTGATinsCTCCTTTCCTTGTG	/	p.207_209delDTDinsAPFLV	Deletion/Insertion mutation	Italian	No	Europe	(27)
Intron 7	c.982 + 5G > T	/	p.Met328Serfs41X	Splice	Japanese	No	East Asia	(51)
Intron 7	c.982 + 2 T > A	/	p.Gly296Val	Splice	Japanese	No	East Asia	(52)
Intron 7	c.982 + 7_ + 21del	/	p.Tyr297_Met328delel	Splice	Japanese	No	East Asia	(52)
Intron 8	c.1095-3C > G	/	p.Glu366Ilefs*7	Splice	Japanese	No	East Asia	(53)

*indicates a nonsense mutation that results in a premature termination codon.

**Table 3:** Reported *LPAR6* gene mutations in patients with ARWH.

Mutation	Variation ID/Accession (ClinVar)	Amino acid changes	Variant type	Ethnicity or geographical area	Founder/Recurrent	Region	Reference
c.436G > A	1828/VCV000001828.4	p.Gly146Arg	Missense	Pakistani, Iranian	Yes	South Asia, Middle East	(54, 55)
c.47A > T	/	p.Lys16Met	Missense	Pakistani	No	South Asia	(54)
c.734A > G	/	p.Tyr245Cys	Missense	Pakistani	No	South Asia	(49)
c.66_69insCATG	/	p.Phe24HisfsX28	Frame-shift	Pakistani	Yes	South Asia	(56)
c.160insA	/	p.N54TfsX58	Frame-shift	Pakistani	No	South Asia	(57)
c.36insA	/	p.D13RfsX16	Frame-shift	Pakistani	No	South Asia	(57)
c.565G > A	1829/VCV000001829.3	p. Glu189Lys	Missense	Pakistani	No	South Asia	(57)
c.8G > C	2,505,339/VCV002505339.1	p.Ser3Thr	Missense	Pakistani	No	South Asia	(57)
c.188A > T	217,499/VCV000217499.3	p. Asp63Val	Missense	Pakistani	Yes	South Asia	(56)
c.562A > T	1827/VCV000001827.4	p.Ile188Phe	Missense	Pakistani	Yes	South Asia	(56)
c.69insCATG	/	p.24insHfs52	Frame-shift	Pakistani	Yes	South Asia	(49)
c.68_69insGCAT	/	p.Phe24Hisfs*29	Frame-shift	Pakistani	No	South Asia	(39)
c.409 T > C	/	Not reported	Missense	Pakistani	No	South Asia	(49)
c.410-426del17	/	Not reported	Deletion	Pakistani	No	South Asia	(49)
c.373_374delAA	/	p.Lys125fs	Deletion	Saudi, Arab	No	Middle East	(58)
c.472delC	/	p.His158ThrfsX27	Deletion	Turkish	No	Middle East	(59)
c.64_67dupTGCA	/	p.Phe24HisfsX28	Duplication	Indian	No	South Asia	(59)
c.669_672delCAAA	/	p.Asn223LysfsX7	Deletion	Israeli	No	Middle East	(60)
c.756 T > A	/	p.Tyr252*	Nonsense	Japanese	No	East Asia	(61)
c.736A > G	3,768,194/VCV003768194.1	p.Cys246Ser	Missense	Spanish	No	Europe	(62)
c.859G > C	/	p.Asp287His	Missense	Spanish	No	Europe	(62)
Not reported	/	p.C278Y	Missense	Brazilian	No	South America	(63)
c.377C > G	/	p.Thr126Ser	Missense	Chinese	No	East Asia	(64)
c.328-330delATT	/	p.Ile110del	Deletion	Chinese	No	East Asia	(65)

**Table 4:** Reported *C3ORF52* and *KRT25* gene mutations in international patients with ARWH.

Causative gene	Mutation	Variation ID/Accession(ClinVar)	Amino acid changes	Variant type	Ethnicity or geographical area	Founder/Recurrent	Region	Reference
*KRT25*	c.712G > T	242,934/VCV000242934.5	p.Val238Leu	Missense	Russian	No	Europe	(15)
*KRT25*	c.950 T > C	217,303/VCV000217303.3	p.Leu317Pro	Missense	Pakistani	No	East Asia	(16)
*C3ORF52*	c.492 T > A	1,806,468/VCV001806468.1	p.Tyr164Ter	Nonsense	Hispanic American	No	North America	(17)
*C3ORF52*	c.34G > T	1,806,469/VCV001806469.2	p.Glu12Ter	Nonsense	Arab Muslim ancestry American	No	North America	(17)

Notably, the frequency of pathogenic gene mutations in ARWH patients varies by ethnicity and geographical region. *LIPH* mutations have been most frequently reported in Pakistan, Japan, China, and Russia, as well as in Italy, India, Austria, Lebanon, Arabia, Jewish populations, and Guyana. High incidences of *LIPH* mutations in ARWH pedigrees have been observed in populations from Japan, Pakistan, and the Volga-Ural region of Russia. In the Japanese population, *LIPH* mutations predominantly occur in exon 6: the c.736 T > A (p.Cys246Ser) variant is the most frequently reported, followed by c.742C > A (p.His248Asn), which are recognized as the primary genetic etiologies. Missense and splice site mutations are the dominant types, and there are numerous cases of compound mutations involving two exons or one exon combined with one intron. In Pakistan, all reported *LIPH* mutations are homozygous, primarily consisting of deletion variants in different exons. Over half of Pakistani ARWH families carry the exon 5 deletion mutation c.659_660delTA (p.Ile220Argfs*29). In the Volga-Ural region of Russia, most ARWH families harbor the exon 4 deletion c.527_628del. In China, 19 ARWH cases have been reported to date, all of which involve *LIPH* mutations. Among these, patients from 12 unrelated families carry either a homozygous c.742C > A (p.His248Asn) mutation or a compound heterozygous genotype consisting of c.742C > A and another functionally deficient variant. The second most common mutation is c.736 T > A (p.Cys246Ser), consistent with the mutation spectrum observed in the Japanese population. Additional genetic testing data are required to confirm potential founder mutations in the Chinese population.

*LPAR6* mutations in ARWH pedigrees have been reported in Pakistan, Saudi Arabia, Iran, Türkiye, India, Israel, Japan, Spain, Brazil, and China; however, a high incidence of *LPAR6* mutations has been documented only in the Pakistani population, with 14 distinct variants identified. Missense mutations are the predominant type, and no high-frequency recurrent variants or confirmed founder mutations have been identified. Domestically, *LPAR6* mutations have only been detected in two ARWH cases.

In 2016, Nikolay V et al. collected data from 119 individuals presenting with hypotrichosis during a field survey in the Volga-Ural region of Russia. In a group of patients who displayed a phenotype similar to conventional hypotrichosis but had some differences, a previously unreported homozygous mutation c.712G > T was detected within the *KRT25* gene, resulting in the p.Val238Leu substitution in the K25 protein. This gene encodes type I (acidic) keratin. Furthermore, the *KRT25* mutation c.712G > T was found in isolated populations in the Volga-Ural region, suggesting a founder effect (15). Additionally, Ansar et al. (16) reported a homozygous K25 missense mutation (p.Leu317Pro) in two unrelated Pakistani ARWH families.

In 2020, Malki et al. first identified homozygous variants in *C3ORF52* in four individuals with LAH and discovered that *C3ORF52* is coexpressed with lipase H in the inner root sheath of the hair follicle, with these two proteins directly interacting. They proposed that lipase H, *C3ORF52*, and *LPAR6* are equally important for normal hair growth. Moreover, PA and LPA have been shown to promote the formation of mouse hair follicles, making these molecules potential targets for the treatment of LAH and other hair disorders (17).

### 3.5 Treatments for isolated ARWH

ARWH treatment remains challenging, though some patients experience gradual hair improvement with age (18). Currently, no sufficiently effective standardized treatment exists.

#### 3.5.1 Evidence-supported options

Topical minoxidil (1, 2%, or 5% solutions/gels) is applied to the affected scalp areas once or twice daily. Studies show that four Japanese ARWH patients with *LIPH* mutations achieved significant hair growth with 1% or 5% minoxidil over 6 months to 3 years, with no adverse effects (19). Note that minoxidil is labeled for androgenetic alopecia; its use for *LIPH*-associated ARWH and congenital hypotrichosis is off-label.

#### 3.5.2 Emerging/experimental options

Gentamicin’s therapeutic value was first validated in hypotrichosis simplex of the scalp: *in vitro* experiments showed it induces read-through activity targeting CDSN mutations, restoring partial protein function and improving hair symptoms (20). Theoretically, it could benefit ARWH patients with similar nonsense mutations, but no direct clinical or *in vitro* data confirmed efficacy for ARWH-related mutations.

Regenerative medicine techniques such as platelet-rich plasma therapy (PRP), human hair follicle stem cells (HFSCs), microneedling, and low-level laser therapy (LLL-T) may promote hair regeneration (21). Our team previously reported safe and satisfactory efficacy of a combined traditional Chinese medicine (TCM) regimen: topical “Hair Growth Tincture” (a China-Japan Friendship Hospital-developed external preparation containing Psoraleae Fructus, Rhododendri Mollis Flos, Zingiberis Rhizoma Recens, and 75% ethanol) plus oral paeoniflorin capsules and compound glycyrrhizin tablets (21). However, the majority of studies are small-sample case reports, with unconfirmed efficacy stability and reproducibility.

With the advancing research on the LIPH/LPA/P2Y5 signaling pathway and ARWH pathogenesis, targeted drugs regulating this pathway may become potential treatments.

## 4 Conclusion

From the existing studies summarized, a preliminary understanding of ARWH, including its clinical features, genetic patterns, and potential treatments, has been established, yet a clear gap remains between mechanistic research and clinical practice. Genetically, while region-specific mutation patterns of genes such as *LIPH* and *LPAR6* (e.g., the high prevalence of *LIPH* c.742C > A in the Chinese population) highlight genetic founder effects, the link between specific mutations and clinical phenotypes (e.g., hair sparseness severity and age-related changes) remains unclear. This hinders genotype-based disease progression prediction and personalized clinical intervention.

Treatment-related limitations are more notable. Topical minoxidil’s efficacy in *LIPH*-mutated patients is only supported by small-sample, short-term observations, with its long-term safety and applicable mutation range unconfirmed. Gentamicin’s theoretical value, based on nonsense mutation correction, lacks validation in ARWH patients, leaving its effectiveness across mutation backgrounds unknown. Meanwhile, TCM compounds and regenerative therapies lack standardized protocols and controlled studies, making efficacy reproducibility hard to assess. As a result, current treatments remain “symptomatic trials” without evidence-based standardized regimens.

Additionally, although the diagnostic system emphasizes the integration of “clinical manifestations-microscopic examination-genetic testing,” practical application faces challenges, including ambiguous terminology and inconsistent gene variant classification standards. These issues reduce data comparability across studies and may cause misdiagnosis or missed diagnosis. Future research should focus on three priorities: clarifying genotype–phenotype associations to build genotyping-based prognostic models; conducting multi-center, large-sample trials to validate existing treatments and explore targeted drugs for pathways such as LIPH/LPA/P2Y5; and establishing unified diagnostic terminology and standards to enable integrated data analysis, ultimately bridging the gap from ARWH mechanistic research to standardized clinical management.

As a review, this study has limitations: (1) No pre-defined protocol leads to subjective bias in literature screening, potentially omitting negative results or regional studies; (2) restricted to CNKI, PubMed, and pre-December 2024 publications, missing non-Chinese/English or non-mainstream database studies; (3) included evidence consists mostly of short-follow-up case reports or series (lacking high-quality data such as randomized controlled trials), with publication bias overstating treatment efficacy; (4) inconsistent ARWH terminology, diagnostic criteria, and variant classification reducing data reliability; (5) treatments rely on off-label use or extrapolation from other diseases, lacking direct ARWH evidence; and (6) genetic data, affected by region/founder effects, lack quantitative analysis, limiting representativeness of global ARWH genetic features.
